# Physiological and pathological consequences of cellular senescence

**DOI:** 10.1007/s00018-014-1691-3

**Published:** 2014-07-31

**Authors:** Dominick G. A. Burton, Valery Krizhanovsky

**Affiliations:** Department of Molecular Cell Biology, The Weizmann Institute of Science, 76100 Rehovot, Israel

**Keywords:** Aging, Age-related disease, Immune surveillance, DNA damage response

## Abstract

Cellular senescence, a permanent state of cell cycle arrest accompanied by a complex phenotype, is an essential mechanism that limits tumorigenesis and tissue damage. In physiological conditions, senescent cells can be removed by the immune system, facilitating tumor suppression and wound healing. However, as we age, senescent cells accumulate in tissues, either because an aging immune system fails to remove them, the rate of senescent cell formation is elevated, or both. If senescent cells persist in tissues, they have the potential to paradoxically promote pathological conditions. Cellular senescence is associated with an enhanced pro-survival phenotype, which most likely promotes persistence of senescent cells in vivo. This phenotype may have evolved to favor facilitation of a short-term wound healing, followed by the elimination of senescent cells by the immune system. In this review, we provide a perspective on the triggers, mechanisms and physiological as well as pathological consequences of senescent cells.

## Overview

**Fig. 1:**
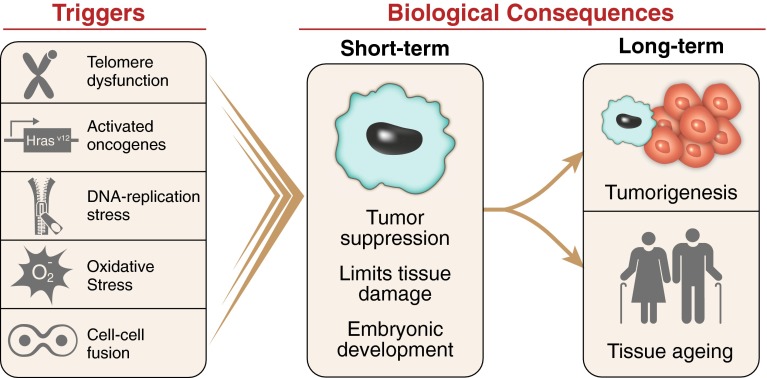
Biological consequences of cellular senescence. Cellular senescence can be induced by various triggers, including, but not limited to, telomere dysfunction, oncogene activation, reactive oxygen species and cell–cell fusion. Short-term presence of senescent cells plays a beneficial role in tumor suppression, wound healing and embryonic development. However, the long-term persistence of senescent cells in tissues can paradoxically promote tumorigenesis and development of age-related diseases

In response to cellular stress, often resulting in DNA damage, proliferating cells can initiate a program that leads to a permanent cell cycle arrest termed cellular senescence. The short-term induction of cell senescence has beneficial roles in tumor suppression, wound healing and possibly embryonic development (Fig. 1). However, the long-term presence of senescent cells in tissues has the potential to promote age-related disease and cancer in a cell in non-autonomous manner. In this review, we discuss the various triggers and mechanisms of cell senescence, the physiological and pathological consequences of the senescence program, the ability of senescent cells to interact with immune cells and provide possible explanations for why senescent cells may persist in tissues.

## Triggers and molecular pathways of cell senescence

Cell senescence can be induced by various stimuli, all of which engage similar molecular pathways to initiate and sustain the senescence program (Fig. 1). The first experimental evidence for the existence of such a program was provided by Hayflick and Moorehead [1] more than 50 years ago. They demonstrated that cultured cells have a maximum limit on the number of cellular divisions that can be undertaken. This replicative limit is a result of the gradual shortening of telomeres due to an inability of replicative polymerases to synthesize DNA at chromosome ends [2, 3]. Chromosome ends consist of a telomere end complex made up of telomeric proteins that protects chromosome ends from being recognized as a double strand break, thereby preventing a DNA damage response (DDR). However, when a telomere becomes critically short, it can no longer be protected and induces a DDR that triggers cell senescence, referred to as replicative senescence (RS) [4]. Telomere elongation by telomerase, an enzyme that adds telomeric repeats back to chromosome ends, protects cells from RS [5, 6].

In addition to RS, cell senescence can be initiated by other stimuli that prematurely induce cell senescence independent of telomere length. The activation of oncogenes such as *RAS* [7] and *RAF* [8] also induces cell senescence, referred to as oncogene-induced senescence (OIS). This form of cell senescence is associated with tumor suppression. A recent genomic study on the comparison of RS cells and OIS cells show that while there are some common gene expression changes between RS and OIS compared to proliferating cells, there are also substantial differences [9]. Although initially limited to in vitro studies, numerous findings suggest that OIS might be mediated, at least partially, by the induction of DNA damage, often associated with elevated reactive oxygen species (ROS) levels [10–14]. Activation of ERK has also been shown to be required for Ras-induced senescence by promoting the degradation of proteins required for cell cycle progression [15]. It also appears that cell replication is required to activate a DDR via oncogene activation, since oncogene expression does not trigger a DDR in the absence of DNA replication [11]. However, the contribution of DDR to OIS in vivo is not completely understood and requires further characterization. Moreover, mutant oncogenes, for example *H*-*ras* ^*G12V*^, have the potential to activate molecular pathways of cell senescence such as p38 and NF-kB independent of DNA damage. In addition, oncogenic Ras can promote the up-regulation of p53 via p19ARF independent of DNA damage in mice [16]. Therefore, the induction of cell senescence in the absence of DNA damage cannot be excluded [17].

DNA damage induced by ionizing radiation, UV light, chemotherapeutic drugs and pathological increases in intracellular and extracellular ROS can also activate the senescence program. This type of cell senescence is often referred to as stress-induced premature senescence (SIPS) [18], because it occurs independent of telomere length, similar to OIS. The induction of SIPS is completely dependent upon a DDR. As with OIS, phenotypically SIPS and RS appear to be similar in many ways, but it has been shown that they can differ at the level of protein expression [19]. Age-related impairment in mitochondrial and antioxidant enzyme systems may lead to an increase in ROS-related damage [20]. Various forms of ROS such as hydrogen peroxide, superoxide and hydroxyl radicals can inflict DNA damage, particularly at telomeres potentially leading to induction of SIPS [18, 21]. Whether SIPS plays a role in normal physiological aging is still debatable, as increased expression of several antioxidant enzymes did not extend the lifespan in mice [22].

In many cases, the above-mentioned triggers of cell senescence lead to the activation of a DDR. It is known that while mild DNA damage can induce a transient growth arrest and extensive DNA damage can induce programmed cell death, persistent DNA damage induces cell senescence [23]. The molecular determinants that regulate the switch from transient growth arrest to irreversible growth arrest are becoming more complex and have yet to be fully determined. However, in general terms, the induction of DNA damage initially activates the p53–p21 pathway to facilitate cell cycle arrest [24]. This pathway is the main driving force for induction of the senescence program. When DNA damage cannot be resolved, p16(INK4a) appears to regulate the long-term maintenance of permanent cell cycle arrest by induction of chromatin changes through Rb pathway [7, 25, 26]. However, cell senescence can also occur independent of p53 and p21 in the presence of DNA damage, which appears to be dependent upon p16(INK4a) [27, 28]. The factors determining why cells enter senescence through the p53/p21 pathways or via p16(INK4a) independent of p53/p21 are not fully understood. It can be suggested that they may be related to the type of initiating stimulus, the extent of DNA damage, cell type-specific differences in the initial levels of p16(INK4a) or the ability to induce its expression. The presence of DNA damage and subsequent up-regulation of p16(INK4a) in quiescent cells in vivo may also induce a pre-senescent state that converts to a full senescent state when cells are stimulated to proliferate [29]. This suggests that DNA replication is required to induce a persistent DDR associated with cell senescence. In vitro, a conversion from transient cell cycle arrest induced by experimental overexpression of p21 to permanent cell cycle arrest has also been described and appears to be dependent upon continuous mTOR activation in response to growth factors [30]. While the induction of cellular senescence via DNA damage is irreversible, induction of long-term cell cycle arrest via p21 overexpression may be reversible if mTOR is inhibited [30].

The induction of persistent DNA damage signaling is not only necessary for induction of permanent cell cycle arrest, but it also facilitates the secretion of soluble factors, including pro-inflammatory cytokines, growth factors and proteases [23]. The secretion of these soluble factors in senescent cells appears to be regulated through p38 and NFκB pathways [17, 31, 32]. Although a number of these secretory factors may be specific to senescence of certain cell types or the method of senescence induction, the commonly secreted factors are often referred to as the senescence-associated secretory phenotypes [33]. An alternative description of this secretory response would be a DNA damage-induced secretory response, which is not limited to senescent cells and may occur in some circumstances in non-senescent cells such as cancer cells or post-mitotic cells [34, 35]. However, since secretory factors may be initiated independent of DNA damage, further research should determine which senescent secretory factors are specific to DNA damage and which are not.

The main molecular pathways of senescence, persistent DDR and Rb lead to sustained chromatin remodeling within senescent cells [26, 36, 37]. This stochastic remodeling of chromatin in senescent cells most likely facilitates promiscuous gene expression associated with cell senescence. Promiscuous gene expression is often observed in microarray data and other analysis of gene expression of senescent verses their non-senescent counterparts and appears to also be cell type specific [38–41]. Promiscuous gene expression refers to changes in gene expression not normally associated with non-senescent counterparts of the same cell type. Chromatin rearrangement would allow access to DNA normally tightly packed and restrict other areas of chromatin that are normally open. It has also been suggested that DNA damage may modulate gene expression by altering the binding capacity of transcription factors [42]. In addition, changes in DNA methylation associated with cell senescence may also contribute to promiscuous gene expression [40, 43]. Therefore, genes that may normally be expressed can be suppressed and genes normally suppressed become expressed.

Cell senescence can thus be defined by three prevalent features which might be associated with a persistent DDR in the majority of cell types: (1) irreversible growth arrest and activation of molecular pathways leading to this arrest, (2) secretion of soluble factors, and (3) promiscuous gene expression. However, it should be emphasized that while different triggers appear to induce a similar senescence response, the molecular differences as a result of the various stimuli are not yet fully understood. The majority of the early work on cell senescence was carried out on fibroblasts in vitro, with a limited understanding of the senescent phenotype of other cell types, especially those linked to age-related pathologies in vivo. Therefore, further investigations are required for detailed characterization of senescent cells in these conditions.

**Fig. 2:**
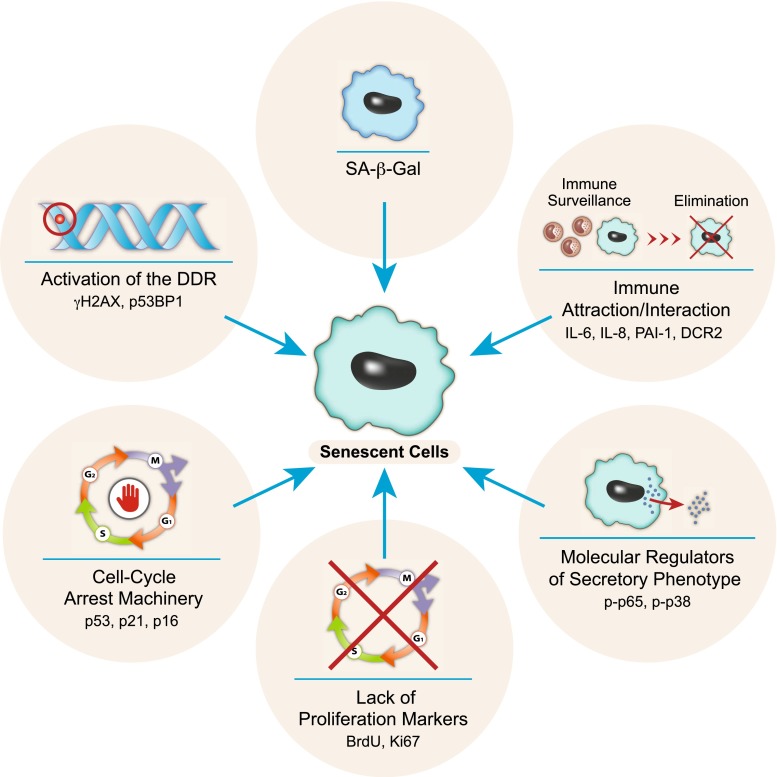
Identifying senescent cells. The use of several *molecular markers* that represent different characteristics of cell senescence is necessary for identifying senescent cells. The markers are divided into categories according to their function. A combination of *markers* representing different categories might increase the validity of the identification

Since cell senescence can be induced in a variety of different cells types and by numerous triggers in different locations within organisms, it is necessary to standardize the set of criteria required to identify senescent cells in tissues. The standard SA-β-gal staining [44], while indicative of the presence of senescent cells, is not an absolute marker for senescent cell and indicates increased lysosomal b-galactosidase activity [45]. The use of several molecular markers that represent different characteristics of senescent cells is necessary (Fig. 2). Such molecular markers can represent the cell cycle arrest machinery (e.g. p53, p21, p16), lack of cellular proliferation (e.g. lack of BrdU incorporation, Ki67), activation of the DDR (e.g. γH2AX or p53BP1 foci), expression of secretory factors (e.g. IL-6 and IL-8), the activation of the pathways that regulate the secretory phenotype (e.g. p-p65 or p-p38), the activation of immune surveillance-related genes and possible regulators for their pro-survival response (DCR2, p-Akt, p-Erk). Loss of Lamin B1 in senescent cells has also been suggested to be a marker of cell senescence [46]. The presence of several such markers in addition to the SA-β-gal should clearly indicate the presence of senescent cells.

## Physiological impact of cell senescence in vivo

### Tumor suppression

While the history of research on cell senescence counts for more than half a century, only in the last 10 years the functional relevance of cell senescence in vivo was established. The irreversible cell cycle arrest in OIS cells makes it an ideal mechanism to prevent tumor formation following oncogene activation [7], and in the first functional in vivo studies, cell senescence was established as a tumor suppressor mechanism [47–50]. OIS has been shown to be important for preventing lymphoma development and contribute to response to therapy [47, 51]. Using transgenic mice models to bypass the senescence response to oncogenic N-Ras resulted in the development of invasive T cell lymphomas, whereas control mice only develop non-lymphoid neoplasia at a much later time point [47]. Another mouse model using inducible K-ras was used to make pre-malignant lesions that can develop into malignant tumors in lung and pancreas [49]. In these models, biomarkers of cell senescence were predominantly identified in the pre-malignant lesions but were lost once tumors developed. To investigate OIS in vivo, a number of studies have focused on human nevi (moles), which are benign tumors of melanocytes that frequently harbor oncogenic mutations of BRAF. The congenital nevi stained positive for markers of OIS, but not DNA damage in this instance. Braf^E600V^, which is present in the nevi, induced p16(INK4a) expression in growth-arrested melanocytes both in vitro and in situ [50]. In contrast, another study in pre-malignant melanocytic lesions did show the presence of DNA damage foci, primarily located at telomeric regions as well as the p16(INK4a) expression [52]. In addition to activating mutations in oncogenes, cell senescence can be induced as a result of loss of tumor suppressor Pten in the prostate [48]. Therefore, these combined studies clearly demonstrate that cell senescence acts as a potent tumor suppressor mechanism that prevents the development of multiple malignancies.

### Limiting tissue damage

In addition to their tumor suppression function, senescent cells also play a beneficial role in non-cancer pathologies by limiting tissue fibrosis [53]. For instance, tissue damage within the liver stimulates the activation of hepatic stellate cells (HSCs), which hyper-proliferate and secrete extracellular matrix components to form a fibrotic scar. Hyper-proliferation of HSCs induces cell senescence leading to a reduction in the secretion of ECM proteins and enhanced secretion of ECM degrading proteins, thereby limiting fibrosis. Senescent HSCs are then eliminated in a timely manner by immune cells such as natural killer (NK) cells. When the mechanisms leading to NK cell-mediated elimination are disabled, fibrosis is increased [54]. In mice lacking molecular components required for induction of cell senescence, HSCs continue to proliferate, depositing ECM components and elevating the fibrotic response [53]. Therefore, induction of senescence in HSCs prevents short-term tissue damage by limiting fibrosis. In addition to the liver, a similar process occurs during tissue repair within the pancreas by senescent pancreatic stellate cells [55]. In this instance, it was suggested that lymphocytes at the sites of wounds might play a duel-specific role in pancreatic fibrogenesis by triggering both the initiation of wound healing by activating stellate cells and its completion by clearance of senescent stellate cells.

Cell senescence also limits tissue damage at sites of cutaneous wound healing, where secretion of CCN1 induces fibroblast senescence associated with an elevation in the DNA damage response and the activation of p53 and RAC1–NOX1 complex [56]. The expression of anti-fibrotic genes by CCN1-induced senescent cells prevented excess fibrosis, whereas mice that express a senescence-defective CCN1 mutant resulted in elevated fibrosis. CCN1 also appears to play a role in the regression of liver fibrosis through induction of cell senescence in HSCs [57]. Therefore, cell senescence is a mechanism that limits tissue damage in multiple tissues and serves not only to restrain the damage, but also to initiate the repair and return the tissue to its pre-damaged state.

### Promoting embryonic development

In addition to providing a protective role in tumour suppression and tissue damage, senescent cells may also function in embryonic development. It was suggested that cell–cell fusion induced senescence (FIS) might play a physiological function in the placenta, thereby aiding embryonic development [58]. ERVWE1, a fusion protein involved in the formation of the syncytiotrophoblast of the placenta causes cell fusion and induction of cell senescence in both cancer cells and normal fibroblasts [58]. FIS in vitro and in vivo is accompanied by activation of a DDR, p53 and p16(INK4a) dependent pathways. ERVWE1 mediated physiological cell fusion during embryonic development forms the syncytiotrophoblast that serves as the maternal/fetal interface at the placenta. The question of why the senescence program may be useful in normal placental function remains to be answered. However, it can be suggested that the resistance of senescent cells to apoptosis [59] is necessary to maintain the viability of the syncytiotrophoblast. In addition, secretion of proteases, that are normally associated with senescent cells, may function to maintain feto-placental homeostasis. Placental proteases are required for the metabolism of vasoactive and immunomodulating peptides, thereby controlling the exchange of peptide hormones across the placenta and metabolic breakdown of maternal nutrients [60]. Cytokine production is another feature of senescent cells that may play important roles within the placenta [61]. IL-8, one of the main cytokines secreted by senescent cells, is necessary for normal placental function [62, 63]. Cytokine secretion may help regulate placental growth during pregnancy [61] in addition to protecting the fetus from pathological organisms and facilitating interaction with immune cells [62, 64]. Further research is necessary to understand the functional significance of the senescence program in the placenta.

A form of cell senescence associated with development of transient fetal structures has also been recently described [65, 66]. Developmental senescent cells were located throughout the embryo including at sites of the mesonephros and the endolymphatic sac of the inner ear [65] and the apical ectodermal ridge and the neural roof plate [66]. The authors suggested that senescent cells play an important role instructing tissue growth and organ patterning. In addition, since tissue remodeling is actively occurring during embryogenesis via the elimination of cells through programmed cell death, developmental senescent cells may also function to maintain tissue integrity. However, these cells do not display a DDR and are induced independent of p53 and p16(INK4a) expression. Instead, these cells are dependent upon p21, regulated via the TGF-β/SMAD and PI3K/FOXO pathways. Interestingly, these developmental senescent cells also share an expression signature with OIS [66]. Ongoing research into the role of cell senescence in embryonic development will provide further insights into its physiological function under these circumstances.

## Pathological impact of cell senescence

Paradoxically, while the induction of cell senescence can initially have beneficial effects by preventing tumourigenesis and limiting tissue damage, their long-term presence within tissues can potentially promote age-related diseases and potentiate cancer formation [67–71]. There are three main features of senescent cells that allow them to have a detrimental impact on the tissues in which they reside. First, the inability to proliferate alone can potentially impair tissue regeneration, more so if stem or progenitor cells become senescent. Second, cellular dysfunction prevents senescent cells from carrying out their normal physiological functions. Third, and maybe most prominently, by negatively impacting the local microenvironment via cell non-autonomous mechanisms.

### Impairment of tissue regeneration

In response to cell loss through tissue damage, cells can undergo cellular proliferation to generate new tissue. This may be from somatic cells residing within the surrounding tissue, from stem cells residing within the same tissue or from stem cells derived from a distant source, such as the bone marrow [72, 73]. In addition, some mitotic cell types such as satellite cells function to maintain and regenerate post-mitotic cells such as skeletal muscle following damage [74]. Therefore, any loss of replicative potential would impact not only the mitotic fraction of cells, but would also impact the repair of post-mitotic cells. For example, damage to skeletal muscle in normal young mice causes the activation of quiescent satellite cells (adult stem cells), which proliferate and undergo myogenic differentiation required for muscle repair. However, a recent study has shown that in geriatric mice (28–32 months of age), satellite cell activation is impaired and satellite cells instead convert from a pre-senescent state [quiescent cells with high p16(INK4a) expression] to a full senescent state (including a DDR) when stimulated to proliferate in response to injury [29]. As such, the induction of senescent satellite cells with age can impair satellite muscle regeneration. This study suggests that senescent cells may accumulate in late life due to a conversion from quiescence to senescence (termed geroconversion) in response to a requirement for cells to replicate over time to regenerate tissue. In this model, more and more quiescent cells are likely to accumulate DNA damage over the life-time of an organism and are, therefore, more likely to become senescent when induced to proliferate later in life.

The induction of cell senescence in another stem cell compartment, the hematopoietic stem cells, has also been suggested to play a role in reducing stem cells renewal capacity associated with age. The expression of p16(INK4a) was shown to be elevated in hematopoietic stem cells with age, thereby limiting hematopoietic stem cell pools and impairing hematopoietic stem cells repopulation potential [75]. In addition, an age-related increase in p16(INK4a) was shown to reduce islet proliferation associated with an impaired regenerative response, whereas mice lacking p16(INK4a) demonstrated enhanced proliferation and regenerative response [76]. Mice lacking p16(INK4a) were also shown to be partially protected from an age-related decline in the self-renewal potential of neuronal progenitors in the subventricular zone and during neurogenesis in the olfactory bulb [77]. Therefore, p16(INK4a) mediated senescence contributes to the decline in a potential of stem and progenitor cells to regenerate tissues.

### Cellular dysfunction

A negative consequence of promiscuous gene expression in senescent cells is impairment in cellular function, the inability of cells to carry out their designated normal processes. As a result, the accumulation of these dysfunctional cells most likely leads to tissue dysfunction which compromises tissue structure and function, promoting disease. For example, a study using Klotho-deficient mice, which exhibit an accelerated aging-like phenotype, investigated whether preventing cell senescence improves health span in these mice [78]. Plasminogen activator inhibitor-1 (PAI-1) is elevated in Klotho-deficient mice and is a known regulator of cell senescence [78, 79]. Klotho-deficient mice deficient in PAI-1 was reported to delay the induction of cell senescence, extending median lifespan and preserving organ structure and function. Another example of senescent cells that can no longer undertake their normal function might include senescent pancreatic beta cells that have impaired insulin release during diabetes [80] and senescent vascular endothelial cells that display decreased activity of nitric oxide synthase (NOS) [81]. NOS is important for the production of nitric oxide (NO) required for maintaining vascular homeostasis and a decrease in NO production is associated with increased risk of cardiovascular disease [82]. Therefore, understanding of the differences in the phenotype of senescent cells of different cell types in relation to their in vivo function is required to better understand mechanisms of disease development.

### Impact on the microenvironment

Senescent cells have the potential to negatively impact their surrounding microenvironment by secreting soluble factors such as cytokines, growth factors and proteases. A number of studies have demonstrated that soluble factors secreted from senescent cells can facilitate cellular proliferation and tumorigenesis in neighboring cells [83–87]. However, these secretory factors might also contribute to tumor-suppressive macrophage polarization and reinforce cell cycle arrest in normal cells, limiting their proliferative potential [31, 88, 89]. However, while the secretion of soluble factors by senescent cells has been the primary focus in understanding how senescent cells promote tumorigenesis, it appears that direct cell contact of senescent cells with neighboring cells may be a more potent mechanism in promoting tumorigenesis [83]. Therefore, other potential mechanisms for promoting tumorigenesis, particularly those involving direct cell contact should be considered in further studies.

Proteases, such as matrix metalloproteinases (MMPs) and collagenases, secreted by senescent cells are able to cause degradation of the extracellular matrix (ECM) [90–92]. ECM remodeling is an important mechanism in the regulation of cell differentiation [93], maintenance of stem cells niches [94], angiogenesis [95], bone remodeling [96] and wound healing [97]. Therefore, it is not difficult to envision how ECM destruction can compromise the functional integrity of the surrounding tissue, promoting disease. An interesting study has demonstrated that when replicative senescent fibroblasts are grown on ECM produced by proliferating cells, they revert back to a “youthful” phenotype [98]. This study thus demonstrates the importance of maintaining ECM structure for regulating cellular function.

It also appears that cells can develop a cell-type exclusive senescent phenotype that could also compromise tissue function and promote disease. When vascular smooth muscle cells (VSMCs) undergo RS they appear to partially trans-differentiate into an osteoblastic, pro-calcificatory phenotype [41, 99]. It was suggested and later shown that secretory factors from senescent VSMCs may play a role in the development of this pro-calcificatory phenotype via an autocrine/paracrine response [100, 101]. It is also likely that secretory factors specific to senescent VSMCs also play a role in the development of this phenotype. Vascular calcification is a major contributor of cardiovascular disease (CVD), suggesting that senescent VSMCs may play an active role in CVD pathophysiology. Interestingly, delaying cell senescence in an accelerated aging mouse model resulted in significant reduction in ectopic calcification [78]. Understanding how different senescent cell types specifically respond to their secretory phenotype may provide further insight into the pathophysiology of various diseases.

## Further evidence for the presence of senescent cells in vivo

While the majority of studies on RS are focused on in vitro studies, evidence for RS in vivo has also been provided. RS cells were detected within livers with chronic hepatitis, cirrhosis and hepatocellular carcinoma as determined by SA-β-Gal staining and measurement of telomere length [102, 103]. Moreover, mice with decreased telomere length were more susceptible for induction of liver cirrhosis [104], suggesting that telomere shortening and induction of cell senescence can contribute to pathological conditions.

Cell senescence has also been shown to be elevated within the skin of aging primates [105]. This study showed that the number of dermal fibroblasts displaying biomarkers of cell senescence such as telomere damage, p16(INK4a) expression and a DDR is elevated in aging baboons. A number of studies have also reported the presence of senescent cells in vivo without clear differences in telomere length. For example, cell senescence in rat kidneys increases with age, associated with an elevation p16(INK4a), but no significant difference in telomere length was observed [106]. Crypt enterocytes within the intestine appear to become senescent with age independent of telomere shortening, associated with elevated DNA damage as determined by γH2A.X immunocytochemistry [107]. Brain tissue from aged individuals and patients with Alzheimer’s disease (AD) was used to investigate astrocyte senescence [108]. Using p16(INK4a) and matrix metalloproteinase-1 (MMP-1) expression as a biomarker of cell senescence, an elevation in astrocyte senescence was observed compared to fetal controls and non-AD adult controls. An earlier in vitro study by the same group also demonstrated that astrocyte senescence could also be triggered by oxidative stress [109]. Further functional studies using in vivo models will provide a better understanding of the possible role of cell senescence in aging and age-related diseases.

## Immune surveillance of senescent cells

The ability of senescent cells to trigger an innate immune response via the up-regulation of pro-inflammatory cytokines was first suggested to play a role in limiting tumorigenesis [110]. This immune response was later shown to be important in the elimination of senescent stellate cells during liver damage [53]. In natural killer (NK) cell-mediated cytotoxicity, NK cells identify senescent cells by the presence of NKG2D ligands on the membrane of senescent cells [53, 111, 112]. The presentation of these ligands on senescent cells might be mediated by a DDR, which was previously shown to induce their expression [113]. In particular, it appears that the ATM–ATR pathway is important for the up-regulation of NKG2D ligands in response to stress [111]. NK cell-induced cytotoxicity of senescent cells is mediated by granule exocytosis and perforin-mediated death rather than death receptor-induced apoptosis [54]. The perforin-mediated cytotoxicity decreases in humans with age [114], and might, therefore, contribute to accumulation of senescent cells in the organism during aging and in age-related diseases. As discussed, senescent cells are known to accumulate with age and in disease states, suggesting that senescent cells may be evading immune surveillance or their rate of accumulation is greater than the rate of removal or both. It has been advocated that the accumulation of senescent cells with age might be the consequence of an impaired aging immune system [70, 115]. In fact, immune cells can also become senescent [116, 117] and these changes may contribute to impaired elimination of senescent cells. Therefore, strategies to restore an aging immune system are a compelling approach for the elimination of senescent cells and for promoting an increased health span.

A recent study has shown that senescent HSCs can be eliminated by another component of the innate immune system, the M1-like macrophages during liver damage and tumorigenesis in the liver [89]. Secretory factors from senescent HSCs were shown to aid the elimination of these cells by macrophages. In contrast, cells that could not become senescent due to deletion of p53 and were not targeted by macrophages. Therefore, the innate immune system appears to be an initial early barrier that regulates the presence of senescent cells in physiological conditions such as in wound healing.

The elimination of senescent cells by the adaptive immune system has also been demonstrated [118]. OIS hepatocytes were shown to secrete cytokines to evoke an immune response leading to the elimination of senescent cells by CD4(+) T cells, a process which required the action of macrophages. The elimination of senescent hepatocytes was required to prevent the development of liver cancer. This study mentions the attraction of T cells by soluble factors but not the mechanism of senescent cell recognition, an area of research that still needs to be explored. However, there is some indication that RS cells may up-regulate MHC1 expression, possibly via p53 [119, 120]. It can be speculated that MHC1 proteins in senescent cells may function to display senescence-associated antigens similar to cancer cells [121], allowing recognition and elimination by cytotoxic T cells. Further research will provide multiple insights into the mechanisms and consequences of the interaction of senescent cells with the immune system.

## Persistence of senescent cells

When cells become senescent in vitro they often become resistant to apoptotic stimuli in comparison to proliferating cells [59, 122]. Although the molecular mechanisms governing this pro-survival response has yet to be fully elucidated, the inability of senescent cells to stabilize p53 in response to further insults of DNA damage appears to play a role in preventing apoptosis [123]. It can be speculated that if immune cells are necessary for eliminating senescent cells, the pro-survival phenotype of senescent cells may function to favor such elimination. In conjunction with regulating immune ligands and the secretory phenotype, persistent activation of the DDR, particularly double strand breaks (DSBs), may also promote a pro-survival response to facilitate DNA repair [124]. However, if senescent cells are not removed by the immune system, this pro-survival phenotype inadvertently promotes their persistence in tissues. Alternatively, the pro-survival phenotype of senescent cells may be an adaptive response mediated by stresses within the microenvironment to facilitate protection from further stress.

The question still arises as to why senescent cells may favor removal by the immune system rather than undergoing programmed cell death. One plausible explanation could be related to the potential function of senescent cells during cellular repair following tissue damage. During wound healing, senescent cells most likely play a positive role by (1) secreting chemoattractants that recruit and activate immune cells to the site of injury, (2) secrete growth factors to stimulate cellular proliferation required for cellular replacement and protein synthesis and (3) the secretion of proteases to debride damaged tissue. In addition, senescent cells may help to preserve tissue integrity during wound healing. If cells underwent apoptosis, the integrity may be lost. Induction of senescence and not apoptosis preserves tissue structure until such time that non-resident cells from other sources, such as stem cells are present to repopulate the tissue with functional cells. In an orchestrated response, senescent cells would be subsequently eliminated by the immune system when no longer required.

If this is indeed the case then the induction of cell senescence, independent of physical wounding (e.g. RS, OIS, SIPS), may evoke a wound healing-like response. However, it is not known whether the induction of cell senescence in a single cell provides enough stimuli to attract immune cells for its elimination. We have suggested above that senescent cells may accumulate as a result of a failure to eliminate them by an aging immune system, but an additional explanation is that they accumulate with age because a single senescent cell is insufficient to stimulate an immune response on its own.

## Concluding remarks

**Fig. 3:**
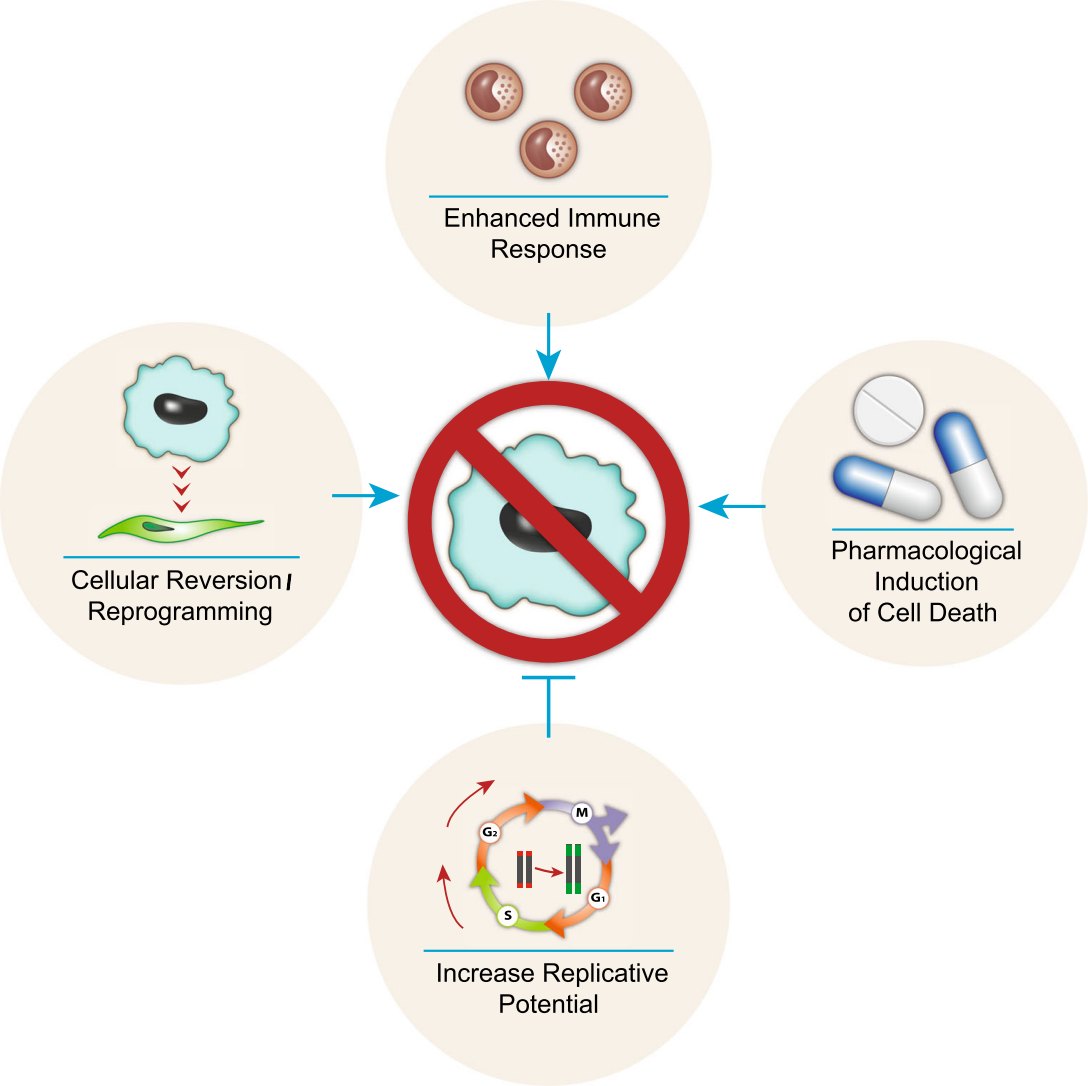
Therapeutic strategies for preventing and eliminating senescent cells. The diagram summarizes the proposed strategies to reduce the presence of senescent cells. These strategies include: extending telomere length with the use of telomerase activators would enhance the replicative lifespan of cells and prevent replicative senescence. Senescent cells could potentially be reverted/reprogrammed back to their functional state. Rejuvenation of an age-impaired immune system may enhance removal of senescent cells. Alternatively, enhancing immune recognition of senescent cells may also be an option. Therapeutic compounds can be developed which specifically target and induce cell death in senescent cells

The persistence and accumulation of senescent cells have been shown to potentially play a role in the pathophysiology of aging and age-related disease. In fact, various disorders associated with accelerated aging such as Hutchinson–Gilford progeria and Werner syndrome have been linked with senescent cells [125, 126]. Therefore, the elimination of senescent cells from tissues has the potential to increase health span and possibly even lifespan. For example, it was recently demonstrated that the elimination of p16-expressing cells in a transgenic mouse model delays age-associated disorders [127]. As such, there are a number of therapeutic avenues of research that have the potential to eliminate senescent cells or prevent their accumulation (Fig. 3). First, telomerase activators could be used to extend telomere length, thereby extending the replicative capacity of cells and preventing RS. Second, cellular reprogramming refers to the potential of reverting senescent cells back to their normal functioning state. Alternatively, if quiescent cells inflicted with DNA damage convert to senescence when stimulated to proliferate, then eliminating such damage may prevent this conversion. Third, if senescent cells indeed accumulate due to failure of removal by an aging immune system, then enhancing the immune response to senescent cells may improve their elimination. Finally, identification of pharmacological compounds that can specifically induce programmed cell death in senescent cells will provide an effective means for elimination of senescence cells regardless the reason of their presence. However, the potential use of future pharmacological compounds should be taken with caution, since senescent cells also play a beneficial role during wound healing. Prematurely eliminating senescent cells during tissue damage may impair the wound response. In fact, it can be speculated that a trade-off may exist between senescent cell removal and wound healing, whereby enhanced senescent cell removal (and possibly slowing aging) results in a slower healing process (and increase risk of infectious disease). Future research will show if pharmacological elimination of senescent cells is a good avenue for treatment of age-related disorders and health span extension.
